# Evaluation of Fiber Post Adhesion to Root Dentin Achieved with Different Composite Cements: 1-year In Vitro Results

**DOI:** 10.3290/j.jad.b2838131

**Published:** 2022-03-24

**Authors:** Uros Josic, Claudia Mazzitelli, Tatjana Maravic, Allegra Comba, Eric Mayer-Santos, Federica Florenzano, Lorenzo Breschi, Annalisa Mazzoni

**Affiliations:** a Research Fellow, Department of Biomedical and Neuromotor Sciences, DIBINEM, University of Bologna, Bologna, Italy; Clinic for Pediatric and Preventive Dentistry, School of Dental Medicine, University of Belgrade, Belgrade, Serbia. Study concept and design, material preparation, wrote first draft, read and approved the final manuscript.; b Adjunct Professor, Department of Biomedical and Neuromotor Sciences, DIBINEM, University of Bologna, Bologna, Italy. Data collection and analysis, wrote first draft, read and approved the final manuscript.; c Junior Assistant Professor, Department of Biomedical and Neuromotor Sciences, DIBINEM, University of Bologna, Bologna, Italy. Material preparation, wrote first draft, read and approved the final manuscript.; d Junior Assistant Professor, Department of Surgical Sciences, University of Turin, Turin, Italy. Data collection and analysis, commented on previous versions of the manuscript, revised it and contributed to the final version of the manuscript, read and approved the final manuscript.; e Adjunct Professor, Department of Restorative Dentistry, College of Dentistry, University of São Paulo, São Paulo, Brazil. Material preparation, wrote first draft, read and approved the final manuscript.; f PhD Student, Department of Biomedical and Neuromotor Sciences, DIBINEM, University of Bologna, Bologna, Italy. Material preparation, read and approved the final manuscript.; g Professor, Department of Biomedical and Neuromotor Sciences, DIBINEM, University of Bologna, Bologna, Italy. Study concept and design, commented on previous versions of the manuscript, revised it and contributed to the final version of the manuscript, read and approved the final manuscript.; h Associate Professor, Department of Biomedical and Neuromotor Sciences, DIBINEM, University of Bologna, Bologna, Italy. Study concept and design, commented on previous versions of the manuscript, revised it and contributed to the final version of the manuscript, read and approved the final manuscript.

**Keywords:** aging, bonding, fiber post, polymerization, composite cement

## Abstract

**Purpose:**

To evaluate push-out bond strength (PBS) and interfacial nanoleakage (NL) of adhesively luted fiber posts using different composite cements and polymerization protocols.

**Material and Methods:**

100 premolars were endodontically treated and assigned to the following groups (n=10): RelyX Universal light-cure (3M Oral Care); RelyX Universal self-cure (3M Oral Care); Maxcem Elite Chroma light-cure (Kerr); Maxcem Elite Chroma self-cure (Kerr); Calibra Universal light-cure (Dentsply Sirona); Calibra Universal self-cure (Dentsply Sirona); Multilink Automix light cure (Ivoclar Vivadent); Multilink Automix self-cure (Ivoclar Vivadent); Luxacore Z Dual light-cure (DMG); Luxacore Z Dual self-cure (DMG). Half of the teeth from each group were subjected to the PBS test after 24 h (T0), while the other half was tested after 12 months (T12) of artificial saliva aging. An additional 4 teeth per group were prepared for NL expression. PBS values were analyzed using multivariate ANOVA and Tukey’s post-hoc test. NL scores were analyzed using chi-squared tests (α = 0.05).

**Results:**

Statistical analysis revealed that the variables “cement” and “aging” significantly influenced PBS (p < 0.05), but not “polymerization” and “root region” (p > 0.05). Significantly lower PBS values (p < 0.05) were detected for the Calibra Universal groups compared to other cements, while the RelyX Universal groups performed equally well (p > 0.05) or better than other cements (p < 0.05). At T12, PBS values increased in the majority of groups, irrespective of root region (p < 0.05). Differences in NL expression were present at T0, and in general, the aging process increased marginal infiltration.

**Conclusion:**

Aging and choice of composite cement influenced PBS, while root region and polymerization protocol seemed to have no influence on posts’ resistance to dislodgment.

Fiber reinforced composite (FRC) posts are commonly used for the restoration of structurally compromised, endodontically treated teeth.^12,20,33^ The complex root canal geometry, limited visibility within the canal, residual material and smear layer created with chemo-mechanical preparation make the cementation of FRC posts challenging.^15^ Composite cements have been considered the material of choice for the cementation of FRC posts.^21^ Conventional, multi-step composite cements rely on the use of adhesives, used in the etch-and-rinse (E&R) or self-etch (SE) mode, to obtain hybridization and intimate adhesion through resin diffusion into the root canal dentin.^4^ The tendency to simplify clinical procedures and reduce operator sensitivity^14^ has led to the introduction of composite cements that could adhere to both the dental substrate and restorations without the need of previous surface treatment.^16,25^ Self-adhesive composite cements have enabled shorter working time due to reduction of clinical steps, but the need to mix all the components (hydrophilic and hydrophobic monomers, catalysts, photoinitiators, etc.) in a single material is a concern, and their use, compared to conventional composite cements, is still a matter of interest in dental research.^27^

Further classification of composite cements is made based on their polymerization modality (light-cure, self-cure, and dual-cure).^31^ In an attempt to overcome the problems related to decreased light transmission in dark areas, such as the apical region of the root canal, dual-cure composite cements were introduced. They were developed by combining the most valuable features of light- and self-cure modalities, providing a certain degree of conversion even in the absence of light.^9^

The topic of composite cement polymerization gives rise to a still-ongoing debate. Dual-cure composite cements contain a mixture of monomers and initiators that do not depend only on light activation to polymerize. Although the chemical activation mode is desirable to contrast the clinical adversities related to the so-called shadow areas,^2,14^ many studies suggest that light-curing of dual-cure composite cements maximizes the polymerization process^6^ and optimizes the mechanical properties of the material.^3^ Since root canal morphology limits the penetration of light into the apical section,^30^ it would be of interest to verify the influence of light-curing on the bond strength of adhesively luted fiber posts, whether they are cemented using conventional multi-step or self-adhesive luting materials.

Therefore, the purpose of this study was to evaluate the push-out bond strength (PBS) and interfacial nanoleakage expression (NL) of composite cements relying on different adhesive approaches (self-adhesive or conventional multi-step) for the cementation of FRC fiber posts at baseline and after 12 months of laboratory storage in artificial saliva. The working hypotheses tested were that PBS (MPa) and the amount of silver grains deposited at the adhesive interface are influenced by: 1. the choice of composite cement; 2. curing mode (light-cure or self-cure); 3. root region (coronal or apical); and 4. artificial aging.

## Materials and Methods

### Specimen Preparation

The study protocol was approved by the Ethics Committee of the Department of Biomedical and Neuromotor Science (DIBINEM), University of Bologna, Italy (protocol N°: 71/2019/OSS/AUSLBO).

One hundred extracted, caries-free mandibular premolars were stored in 0.5% chloramine solution at 4°C for no longer than 2 months after extraction. The teeth were sectioned at the cementoenamel junction perpendicular to the long axis of the tooth, using a low-speed diamond saw (Microremet, Remet; Bologna, Italy) under water cooling. Root canal treatment was performed using Pathfiles (#1-2-3) and ProTaper (S1-S2-F1-F2-F3) (Dentsply Sirona; Konstanz, Germany) until the working length was reached. During instrumentation, the canals were irrigated with 5 ml of 5% sodium hypochlorite (Niclor 5, Ogna; Muggiò, Italy), followed by a final rinse with 1 ml of 10% ethylenediamine tetra-acetic acid (Tubuliclean, Ogna). In accordance with the continuous wave technique, the canals were filled with endodontic sealer (AH-Plus, Dentsply Sirona), medium-sized gutta-percha points with DownPack (Hu-Friedy; Chicago, IL, USA), and warm gutta-percha (Obtura III, Analytic Technologies; Redmond, WA, USA). The coronal entrance of the filled roots was then temporarily sealed with a glass-ionomer cement (Fuji VII, GC; Tokyo, Japan) and the samples were stored for 24 h at 37°C and 100% relative humidity.

### Luting of Fiber Posts

After removing the temporary coronal seal, post space was prepared in a standardized way for each tooth. An 8-mm post space was created by using a low-speed dental handpiece and post drill (RelyX fiber post drill Size 2, 3M Oral Care; St Paul, MN, USA). The root canal was then irrigated with 5 ml of distilled water and dried with absorbent paper points (Dentsply Sirona; Konstanz, Germany). Before the luting procedures, a size- 2 fiber post (RelyX, 3M Oral Care) was inserted into the canal to check if it reached the intended length, after which the coronal part outside the canal was cut with a diamond bur. The teeth were then randomly assigned to one of the following groups, according to the luting agent and polymerization protocol employed (N = 10):

- Group 1a (RXU LC): light-cure RelyX Universal (3M Oral Care);
- Group 1b (RXU SC): self-cure RelyX Universal (3M Oral Care);
- Group 2a (MAX LC): light-cure Maxcem Elite Chroma (Kerr; Orange, CA, USA);
- Group 2b (MAX SC): self-cure Maxcem Elite Chroma (Kerr);
- Group 3a (CAL LC): light-cure Calibra Universal (Dentsply Sirona);
- Group 3b (CAL SC): self-cure Calibra Universal (Dentsply Sirona);
- Group 4a (MUL LC): light-cure Multilink Automix/Multilink Primer (Ivoclar Vivadent; Schaan, Liechtenstein);
- Group 4b (MUL SC): self-cure Multilink Automix/Multilink Primer (Ivoclar Vivadent);
- Group 5a (LUX LC): light-cure Luxacore Z Dual/LuxaBond TotalEtch System (DMG; Hamburg, Germany);
- Group 5b (LUX SC): self-cure Luxacore Z Dual/LuxaBond Total Etch System (DMG);

MAX and CAL are self-adhesive composite cements. RXU is defined as universal composite cement that, in the present study, was used in the self-adhesive modey (no previous application of Scotchbond Universal Plus adhesive). MUL is a composite cement that relies on a self-etch approach (SE). LUX is a core buildup and radicular post luting composite used in combination with an etch-and-rinse (E&R) adhesive. The details of fiber-post surface pretreatments, chemical compositions, and application modes of the cements are shown in Table 1.

**Table 1:** The details of fiber-post surface pretreatments, chemical compositions and application modes of the cements

Composite cement (Lot No.)	Composition	Application mode	FRC post preparation
RelyX Universal, 3M Oral Care; St Paul, MN, USA (VTGHESP0019)	BPA derivative free dimethacrylate monomers, phosphorylated dimethacrylate adhesion monomers, photoinitiator system, novel amphiphilic redox initiator, radiopaque fillers and rheological additives, pigments	Self-adhesive composite cement: dispense into the post space and insert the post.	Clean with alcohol and air dry for 5 s. Priming with adhesive not required for 3M RelyX fiber posts.
Maxcem Elite Chroma, Kerr; Orange, CA, USA (71887933)	HEMA, GDM, UDMA, 1,1,3,3-tetramethylbutyl hydroperoxide TEG-DMA, fluoroaluminosilicate glass, GPDM, barium glass filler, fumed silica (69 wt%)	Self-adhesivecomposite cement: dispense in the post space and insert the post.	Clean with alcohol and air dry for 5 s. Apply a layer of silane coupling agent (Ultradent) for 60 s and gently air dry.
Calibra Universal, Dentsply Sirona; Konstanz, Germany (170821)	UDMA, trimethylolpropane trimethacrylate TMPTMA, bis-EMA (bisphenol A ethoxylate dimethacrylate), TEG-DMA, HEMA, 3-(acryloyloxy)-2-hydroxypropyl methacrylate, urethane modified bis-GMA, PENTA, silanated barium glass, fumed silica (48 vol %)	Self-adhesive composite cement: dispense in the post space and insert the post.	Clean with alcohol and air dry for 5 s. Apply a layer of silane coupling agent (Ultradent) for 60 s and gently air dry.
Multilink Automix, Ivoclar Vivadent; Schaan, Liechtenstein (Y47572)	Dimethacrylate and HEMA, barium glass and silica filler, ytterbiumtrifluoride (68 wt%), catalysts, stabilizers, pigments	Self-etch 1-step adhesive composite cement: Mix Multilink Primer (1:1) and apply with a endobrush to radicular dentin for 30 s. Remove the excess with an absorbent paper point. Dispense the cement into the post space and insert the post.	Clean with alcohol and air dry for 5 sec. Apply a layer of Monobond Plus (Ivoclar Vivadent) for 60 s and gently air dry.
Luxacore Z Dual, DMG; Hamburg, Germany (211108)	Bis-GMA, UDMA, barium glass, colloidal silica, nanocomposite, zirconium dioxide 71% weight	Etch-and-rinse 3-step adhesive composite cement: Apply DMG etching gel for 15 s on radicular dentin, rinse with water for 15 s. Dry the canal with paper points. Work 1 drop of prebond (Luxacore Total Etch) into dentin for 15 s, remove the excess with paper point, gently air dry. Mix Bond A and Bond B (1:1) and apply to dentin surface for 20 s using a microbrush, gently air dry. Dispense the cement in the post space and insert the post.	Clean with alcohol and air dry for 5 s. Apply a layer of silane coupling agent (Ultradent) for 60 s and gently air dry.

One operator unaware of the polymerization protocol performed the fiber-post luting procedures. Then, a second operator randomly assigned the specimens either to the LC or SC groups by means of simple randomization (toss of a coin). Light curing was performed through the fiber post for 60 s with an LED curing lamp (1470 mW/cm^2^, Elipar Deep cure, 3M Oral Care). The SC groups were put in dark chambers for 1 h at 37°C to allow exclusively chemical polymerization of the composite cements.

Afterwards, in order to prevent dehydration,^1,13^ the specimens were wrapped in moist medical gauze, put into plastic chambers, and stored in an incubator at 37°C for 24 h . After storage, each root was sectioned into at least six 1-mm-thick slices using a low-speed diamond saw (Microremet, Remet) under water cooling. The first coronal slices were automatically discarded, the coronal side of each slice was marked with an indelible marker to later ensure exact positioning during testing. Six slices from each root were obtained, with first three slices being considered as coronal, while the last three were apical slices. Half of the specimens from each group (N=5) were immediately processed for PBS testing (T0). The other half was stored at 37°C for 12 months (T12) in 2-ml Eppendorf tubes filled with artificial saliva that was regularly changed every 2 weeks.

### Push-out Bond Strength Test

The thickness of each slice was measured using a digital caliper (Starrett 727, Starrett, Itu, SP, Brazil) with ±0.01-mm accuracy. The slices were then put on 1-mm-square graph paper and photographs were taken with a digital camera (D 7200, Nikon; Tokyo, Japan), after which the coronal and apical diameters of the posts were measured using ImageJ software (National Institute of Health; Bethesda, MD, USA). The push-out test was performed using a universal testing machine (Instron 4465, Instron; Norwood, MA, USA) by applying an axial load at a crosshead speed of 0.5 mm/min. The apical surface of the slice was placed facing the punch tip to ensure that the load was applied in an apical-coronal direction, in order to dislocate the post towards the wider part of the slice. The load that caused specimen failure (manifested by the dislodgment of the post) was recorded in Newtons (N) and converted to MPa by dividing the load in Newtons by the bonded surface area (SL) in mm^2^. The bonded surface area was calculated using the following formula:

SL = (π(R+r))*((h^2^ + (R-r))^2^)0.5,

where R is the coronal diameter of the canal with the post, r the apical diameter, and h the thickness of the slice.^8^

One investigator analyzed the debonded specimens using a stereomicroscope at 40X magnification (Stemi 2000-C; Carl Zeiss; Jena, Germany), and the failure mode was classified as follows: adhesive between dentin and the cement (AD), adhesive between the cement and the post (AP), cohesive within the cement (CC), cohesive within the post (CP), and mixed (M).

### Interfacial Nanoleakage Expression

Additional mandibular premolars (N=4 per group) were used to quantify the interfacial NL expression. Endodontic treatment, fiber post cementation, and cutting procedures were performed as previously described for the PBS test. NL analysis was performed at baseline (T0) and after 12-month (T12) storage in artificial saliva at 37°C. The specimens were prepared and covered with nail varnish, leaving 1 mm free at the interface, then immersed in a 50 wt% ammoniacal silver nitrate solution for 24 h. Specimens were then photodeveloped to reduce the diamine silver ions ([Ag(NH_3_)_2_]^+^) into metallic silver grains. The silver-impregnated specimens were fixed, dehydrated in ascending ethanol solutions, embedded in epoxy resin (Epon 812, Fluka; Buchs, Switzerland) and processed for light microscopy, as described by Mazzoni et al.^18^ Images of the adhesive interfaces were captured (20X magnification) and the extent of interfacial NL was scored by one observer using a four-point scale.^26^ Briefly, interfacial nanoleakage was scored based on the percentage of the adhesive surface showing silver nitrate deposition: 0, no nanoleakage; 1, < 25% with nanoleakage; 2, 25% to 50% with nanoleakage; 3, 50% to 75% with nanoleakage; 4, > 75% with nanoleakage.^26^

### Statistical Analysis

After checking the normality (Shapiro-Wilk test) and homoscedastic (modified Levene’s test) assumptions of the data sets, ANOVA was performed to examine the effects of the dependent variables “cement”, “curing mode”, “root region” and “aging”, and the interaction of these factors on PBS. Pairwise comparisons were performed using Tukey’s post-hoc test.

In addition, one-way ANOVA with the post-hoc Bonferroni correction was conducted to evaluate the differences between the groups. NL scores were analyzed using chi-squared tests. All statistical analyses were conducted with the software Stata 12.0 (Stata; College Station, Texas, USA), and statistical significance was set at p < 0.05.

## Results

### Push-out Bond Strength Test

Mean PBS values (MPa) with standard deviations (SD) of specimens tested at T0 or T12 are presented in Tables 2 and 3 for the coronal and apical root regions, respectively. The statistical analysis revealed that the “cement” and “aging” statistically significantly influenced the PBS (p < 0.05), but not the variables “polymerization protocol” and “root region” (p > 0.05). The interactions between the variables were not statistically significant (p > 0.05). The results of the one-way ANOVA demonstrated the trend of statistically significantly lower PBS values in the CAL groups compared to the other cements investigated (p < 0.05). RXU cement performed either as well as (p > 0.05) or better than other self-adhesive and multi-step systems (p < 0.05). This was particularly evident at T12 in SC mode, where RXU showed higher bond strength compared to the other investigated systems, especially in the apical root region (p < 0.05). After artificial aging, PBS values increased in the majority of the investigated groups, irrespective of the root region (p < 0.05).

**Table 2:** Push-out bond strengths ± standard deviations (MPa) in the coronal section at T0 and T12

Groups	T0		T12	
	LC	SC	LC	SC
RXU	16.5p p ± 3.7^bA^	15.0p ± 4.3^bA^	23.3p ± 6.0^aA^	28.9p ± 6.5^aA^
MAX	15.6p ± 4.6^aA^	19.6p ± 3.1^aA^	16.1p ± 6.0^aB^	19.7p ± 8.7^aB^
CAL	8.6p ± 4.5^cB^	14.7p ± 6.1^bAB^	24.5p ± 3.6^aA^	16.4p ± 7.0^bB^
LUX	17.4p ± 5.4^bA^	12.6p ± 3.0^bcB^	30.2p ± 9.5^aA^	21.6p ± 5.9^bAB^
MUL	18.4p ± 6.2^aA^	20.4p ± 7.3^aA^	25.0p ± 8.6^aA^	22.8p ± 6.5^aAB^

Different superscript letters indicate significant differences. Lower-case letters refer to differences within the rows, upper-case letters refer to differences within the columns. LC: light cure; SC: self-cure.

**Table 3:** Push-out bond strengths ± standard deviations (MPa) in the apical section at T0 and T12

Groups	T0		T12	
	LC	SC	LC	SC
RXU	17.7p ± 6.6^bA^	19.9p ± 4.8^bA^	27.7p ± 10.9^aA^	30.3p ± 7.8^aA^
MAX	13.1p ± 7.3^bB^	23.3p ± 3.3^aA^	26.2p ± 5.1^aA^	20.9p ± 7.3^aB^
CAL	5.9p ± 3.9^bC^	8.1p ± 2.6^bB^	16.5p ± 6.3^aB^	14.8p ± 8.9^aC^
LUX	18.7p ± 6.7^bA^	20.4p ± 3.3^bA^	31.4p ± 8.3^aA^	21.6p ± 12.0^bB^
MUL	19.0p ± 4.5^bA^	19.9p ± 6.2^bA^	26.2p ± 10.9^aA^	16.8p ± 5.9^bB^

Different superscript letters indicate significant differences. Lower case letters refer to differences within the rows, upper case letters refer to differences within the columns. LC: light cure; SC: self-cure.

The percentage of the types of failure mode within each group is presented in Table 4. A predominance of mixed and adhesive failures at the cement/post interfaces was observed among the groups, independent of the curing mode and aging conditions. Adhesive failures on the dentin side were observed at T0 for MAX SC, CAL SC, and MUL SC, and at T12 for RXU both in the LC and SC groups. No cohesive fractures were detected.

**Table 4:** Failure mode of the dislodged specimens from five experimental groups at baseline (24 h) and after one year of aging in artificial saliva

Groups	T0		T12	
	LC	SC	LC	SC
RXU	M: 52 AP: 48 AD: 0 CC: 0 CP: 0	M: 60 AP: 40 AD: 0 CC: 0 CP: 0	M: 50 AP: 16.6 AD: 33.4 CC: 0 CP: 0	M: 36.3 AP: 9 AD: 54.7 CC: 0 CP: 0
MAX	M: 70 AP: 30 AD: 0 CC: 0 CP: 0	M: 41.6 AP: 25 AD: 33.4 CC: 0 CP: 0	M: 54.5 AP: 45.5 AD: 0 CC: 0 CP: 0	M: 70 AP: 30 AD: 0 CC: 0 CP: 0
CAL	M: 62.5 AP: 37.5 AD: 0 CC: 0 CP: 0	M: 41.6 AP: 25 AD: 33.4 CC: 0 CP: 0	M: 85.5 AP: 14.5 AD: 0 CC: 0 CP: 0	M: 65 AP: 35 AD: 0 CC: 0 CP: 0
LUX	M: 36.3 AP: 63.7 AD: 0 CC: 0 CP: 0	M: 33.3 AP: 66.7 AD: 0 CC: 0 CP: 0	M: 36.3 AP: 63.7 AD: 0 CC: 0 CP: 0	M: 66.6 AP: 33.4 AD: 0 CC: 0 CP: 0
MUL	M: 55 AP: 45 AD: 0 CC: 0 CP: 0	M: 52.9 AP: 11.7 AD: 35.4 CC: 0 CP: 0	M: 53.3 AP: 46.7 AD: 0 CC: 0 CP: 0	M: 76.9 AP: 23.1 AD: 0 CC: 0 CP: 0

Data are expressed as percentages (%) of the total number of specimens tested for each group. AD: adhesive, between dentin and the cement; AP: adhesive between cement and post; CC: cohesive within cement; CP: cohesive within post; M: mixed.

### Interfacial Nanoleakage Expression

**Fig 1:**
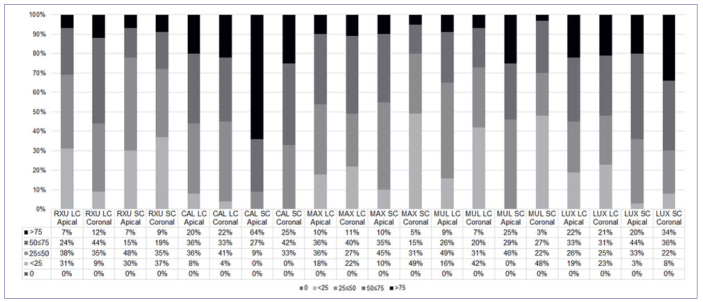
Percentage of interfacial nanoleakage expression in resin-dentin interfaces created in radicular dentin at T0.

**Fig 2:**
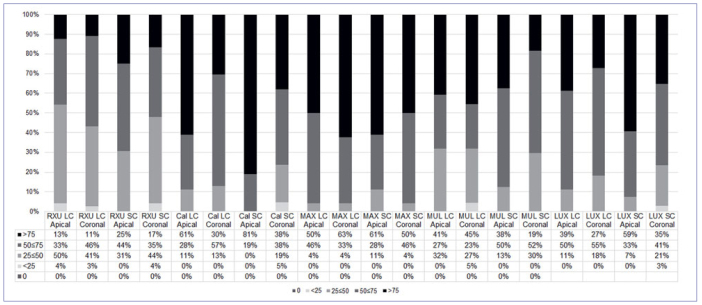
Percentage of interfacial nanoleakage expression in resin-dentin interfaces created in radicular dentin at T12.

Descriptive statistics of interfacial NL scores within the groups in the experimental conditions are presented in Figs 1 and 2 at T0 and T12, respectively. The statistical analysis showed significant differences in the interfacial silver deposition among the tested groups, and this was material-dependent (p < 0.05). At T0, LUX and CAL revealed higher silver nitrate infiltration both in the LC and SC groups (p < 0.05). RXU, MAX, and MUL showed comparable results, independent of the curing protocol performed. Furthermore, no differences were detected between the apical and the coronal portion of the root, except for CAL SC, which exhibited significantly higher NL in the apical portion (p < 0.05).

**Fig 3:**
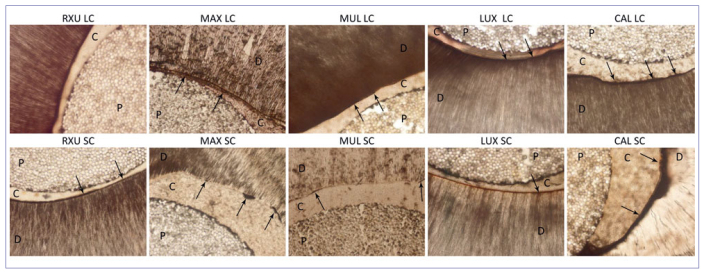
Light micrographs showing the adhesive interface created by different composite cements at T0. Top row: coronal root slices; bottom row: apical root slices. Arrows indicate silver nitrate deposition; D, dentin; P, post; C, cement.

**Fig 4:**
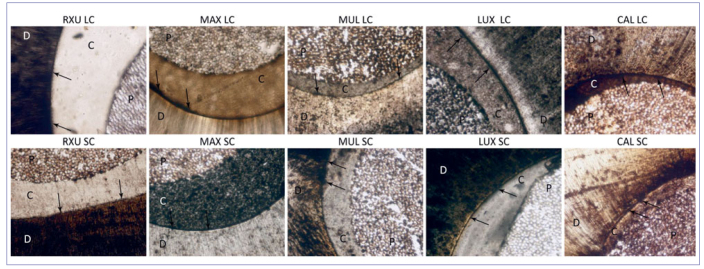
Light micrographs showing the adhesive interface created by different composite cements at T12. Top row: coronal root slices; bottom row: apical root slices. Arrows indicate silver nitrate deposition. D: dentin; P: post; C: cement.

After 12 months of artificial aging, differences in marginal infiltration among the tested groups were still present (p < 0.05). In general, the aging process produced an increase in marginal infiltration, with the following results: MAX = CAL > MUL = LUX > RXU (p < 0.05), irrespective of the polymerization condition and root region. Representative images of NL expression are shown in Figs 3 and 4 (at baseline and after 12 months, respectively).

## Discussion

This study aimed to investigate the bonding performance and sealing ability of different composite cements. According to the results obtained, the first working hypothesis was accepted, since PBS and interfacial NL expression were influenced by the choice of composite cement.

This study used three different bonding strategies for the cementation of FRC posts in root canals. Specifically, LUX and MUL are referred to as multi-step composite cements (E&R and SE, respectively), as the luting procedures require more than one clinical step, whereas RXU, MAX, and CAL rely on a self-adhesive approach, and no pre-treatment of dentin is necessary. Additionally, the new RXU system does not require pretreatment of the post with silane, further simplifying the clinical cementation procedure. In order to optimize the bond strength between fiber post and composite cement, the surface of posts was pretreated differently among the groups, following the manufacturers’ instructions for the specific cement used during luting procedures. However, since a recent systematic review reported that the use of silane alone cannot enhance FRC post resistance to dislodgment,^19^ fiber-post pretreatment was not considered as an additional variable in this study.

Previous studies showed that bonding strategy can influence the hybrid layer appearance and the integrity of the resin-based restorations. Dentin etching with phosphoric acid performed in the E&R approach removes the smear layer, opens the dentinal tubules and reveals the intertubular dentin collagen network, favoring the penetration of the resin to create longer, thicker resin tags and a more uniform hybrid layer than those achieved with the SE approach.^2^ On the other hand, superficial dentin demineralization was observed with self-adhesive composite cements, with very thin, short resin tags.^10^ Although it would seem logical to assume that multi-step composite cements would exhibit a more durable bond strength to root canal dentin compared to the simplified self-adhesive composite cements, the results of the present study emphasize that simplified systems can perform equally well or even better, and that the bond strength is correlated to the cement type. This observation is in agreement with a recent systematic review.^27^

The formation of a reliable and stable bond is in part related to the composite cement polymerization process.^6^ Dual-cure composite cements contain a combination of initiators present in both light-cure and self-cure adhesives, and subsequent photoactivation of dual-cure cement activates photoinitiators and starts polymerization of the material. A proper polymerization reaction of the material translates into better physical and chemical properties,^6^ increased stability and integrity at the adhesive interface,^29^ reduced water sorption/solubility, and greater restoration durability.^24^ In the present study, light curing did not influence the bond strength of the adhesively luted posts, but it did impact the marginal infiltration of some composite cements tested. Consequently, the second working hypothesis could only be partially accepted. This may be explained by the composition of the composite cements used in this study. As the simplified self-adhesive dual-cure cements are expected to achieve surface demineralization of enamel and dentin, they contain acidic monomers.^10^ Despite their important role in the interaction with the cementation substrates, these monomers could lead to the inactivation of the conventional organic polymerization initiators, such as benzoyl peroxide/aromatic tertiary amines, impairing both the chemical and light polymerization process.^20,28^ This particular traditional initiator is present in CAL cement, possibly contributing to the generally poor performance of this material. On the other hand, MAX introduced an amine-free redox initiator, while the new RXU contains a novel amphiphilic redox initiator (ARI system). The new self-adhesive composite cement showed comparable or even superior bond strength both in LC and SC when compared to the other cements tested. According to the claims of RXU’s manufacturer, the ARI system, alongside with functional monomers, enables the cement to diffuse into the smear layer, achieving a strong bond to dentin. Furthermore, the ARI system and functional monomers in the new self-adhesive cement possibly led to the formation of a highly crosslinked 3D polymer network, which is considered to be responsible for the long-term stability of the resin-dentin interface.

The establishment of a fine equilibrium between the different components of the cements, with efficient polymerization initiation and propagation, would be expected to resolve the issue of differences in the quality of polymerization in different root regions. This is in accordance with the present study as well as previously published research,^22^ since the root region did not influence bond strength and NL expression, requiring the rejection of the third working hypothesis.

One-year aging in artificial saliva influenced the bonding performances of the tested materials, which led to the acceptance of the fourth working hypothesis, as the bond strengths and NL expression significantly increased after artificial storage. The exposure of the root slices to artificial saliva for 12 months may have enabled water molecules to enter the composite cement and fiber posts by diffusion.^7,32^ Water diffusion into the material could influence its hygroscopic expansion. The volumetric expansion of composite cement and fiber posts could increase the frictional resistance between the material and canal walls, resulting in its greater resistance towards the axial forces applied during the push-out test.^11^ Interestingly, higher bond strengths were observed for the new self-adhesive RXU cement at T12 compared to the other tested cements. Self-adhesive composite cements show different water sorption and solubility characteristics.^17^ Acidic monomers with hydrophilic characteristics can absorb more water than conventional composites or multi-step composite cements, which would lead to their higher net expansion and more intimate contact to root canal walls.^23^

Although a recent systematic review found considerable variations in the design of the push-out test among studies,^5^ it is considered to be more appropriate and reliable for FRC post testing than microtensile bond-strength tests. Therefore, evaluation of the adhesively luted FRC posts by means of push-out bond-strength tests is irreplaceable in the early screening of dental material properties. Mechanical tests and spectroscopy studies should be performed to better define the mechanical and curing characteristics of the recently introduced self-adhesive universal composite cement, followed by randomized clinical trials.

## Conclusion

Aging and the choice of material influenced the bond strength between adhesively luted fiber posts and radicular dentin. Polymerization protocol and root region had no effect on post retention in the root canal.

## Clinical relevance:

The choice of composite cement, rather than polymerization protocol, influenced fiber post retention in root canals.
